# Nutritional Considerations for Elite Golf: A Narrative Review

**DOI:** 10.3390/nu15194116

**Published:** 2023-09-23

**Authors:** Nicholas Berlin, Matthew B. Cooke, Regina Belski

**Affiliations:** Sport, Performance and Nutrition Research Group, School of Allied Health, Human Services and Sport, La Trobe University, Melbourne, VIC 3086, Australiamatt.cooke@latrobe.edu.au (M.B.C.)

**Keywords:** golf, nutrition, performance, hydration, energy, supplements, travel, diet

## Abstract

Golf is predominantly a skill-based sport where technical aspects are regarded as a priority area for improving performance. At present, most of the existing literature has focused on improving a player’s physicality, endurance and technical attributes in an effort to enhance performance. While important, the role of nutrition in elite golf has received little attention to date. The energy demands of the sport can vary depending on the level of the individual (recreational–professional), with distances of up to 20 km being covered and the time spent on the course ranging approximately 4–8 h each day. Like other sports, a focus on pre-game, during and post-game nutrition, including hydration, is integral to ensuring that individuals are adequately fuelled, hydrated and optimally recovered. For the elite athletes who travel extensively to international tournaments, it is important to understand the additional impact of travel on the body and consider the role nutrition can play in preventing illness and ensuring minimal disruption to golf performance. Lastly, the role of dietary supplements to enhance the performance of golfers is also important to consider. This review aims to consolidate the findings of the existing research focusing on nutrition strategies for golf performance and identify areas for potential future research.

## 1. Introduction

Golf is a game of skill performance and can be played at a recreational level through to an elite level. The Professional Golf Association (PGA) is the most elite international touring circuit with many events held annually. The PGA has many entry points into the tour which competes on both a national and international level. In addition to the PGA, many other tours exist at a professional level worldwide which creates intense travelling schedules for some athletes. These tours include: the LIV Golf tour, DP World Tour (Europe), Korn Ferry tour, Asian tour, Latin American tour, Australasian tour and many more [1]. Players can intertwine between tours which can create a travel schedule across multiple countries and time zones [2,3]. For example, an elite golfer could play a few months on the PGA tour in America and the remainder of the season on the DP World Tour in Europe. Table 1 illustrates the demands of a professional golfer competing in different professional golfing tours for the 2022–2023 season, irrespective of training schedules.

Amateur players can also compete nationally or internationally as some individuals prefer to pursue the ‘best amateur’ rankings before turning professional and thus experience high travel demands as well. The US amateur is a competition which involves 72 holes of stroke play (4 consecutive days), followed by a cut line of the top 32 players who move on to a knockout tournament (one competitor vs. another), until a winner is determined. These tournaments can require an amateur golfer to play up to 36 holes a day and be on the golf course for up to 10 h [1].

To compile this narrative review an electronic database search was conducted. Platforms such as PubMed, Google Scholar and Web of Science were used to identify any of the relevant literature. Search terms included: ‘golf and nutrition/nutrients, or golf and supplements, or golf and hydration’ as well as more generic terms where minimal results for golf were found; for example, ‘nutrition and cognition, or nutrition and performance’. These were utilised to cross reference any relevant information that was deemed useful for performance in cognition and or skill-based sports. Given the scarcity of peer-reviewed papers in this field, two relevant books with content specific to golf performance were also used in this review and additional references were sought from their reference lists.

## 2. Physiological Demands of Golf

Success in golf is seen to be more about the technical, tactical and mental aspects rather than physical attributes as varying physique and fitness levels that golfers exhibit can be of relatively less importance compared to other sports [4]. In fact, the aerobic capacity of golfers is typically in the lower range compared to other more demanding endurance-based sports [5], with reported values in the range from 45.7 mL/min/kg in elite female golfers to 33.8 mL/min/kg in middle-aged amateur golfers, though the latter value included both sexes [6]. An assessment of elite junior golfers on a Wingate test reported peak power values of 722.3 Watts (9.64 W/kg) which shows similar results compared to wrestlers, track and field athletes and football players [6].

The sport requires an athlete to use all energy systems to meet the energy demands, from the explosive nature of the golf swing to the distances covered on the golf course [5]. A typical 18-hole competitive round can take anywhere from 3 to 5 h to complete depending on factors such as the number of players in the group, distance of the golf course, course topology and the accuracy of players, with the number of shots taken during the round [4]. The average length of a golf course is between 7–8 km; however, a golfer can walk up to 10–20 km per round depending on the aforementioned factors [5]. Recently, the PGA and the R&A have lengthened professional golf courses and thus the physical demands are now even greater on golfers.

The entire golf swing lasts less than 2 s, but a tournament professional will perform over 2000 swings during competition and up to 300 powerful movements per practice session. Throughout the initiation of the golf swing, forces are built from the ground upwards, and a large kinetic force is generated through the hips, pelvis and lower back, suggesting that strength in these areas is essential for optimal golf performance [7,8,9]. Studies show that low handicap players have significantly greater rotational strength and one rep maximum strength than high handicap golfers [9]. Finally, environmental factors can also impact the physiological status of the body with temperature, altitude and course topology all affecting internal energy regulation [7].

## 3. Energy Demands of Golf

To adequately fuel and meet the nutritional requirements of a golfer, especially for competitive performance, it is important to understand the energy demands and energy costs of activity [9]. During a winter season, a professional golfer could play in ten tournaments, which equates to approximately fifteen days of competition each month [2]. In addition, players that make ‘the cut’, which refers to the halfway mark of a tournament where the top half of the competitors are identified, play an extra two days (from 2–4 days), and as such, this further increases competitive demands.

**Figure 1:**
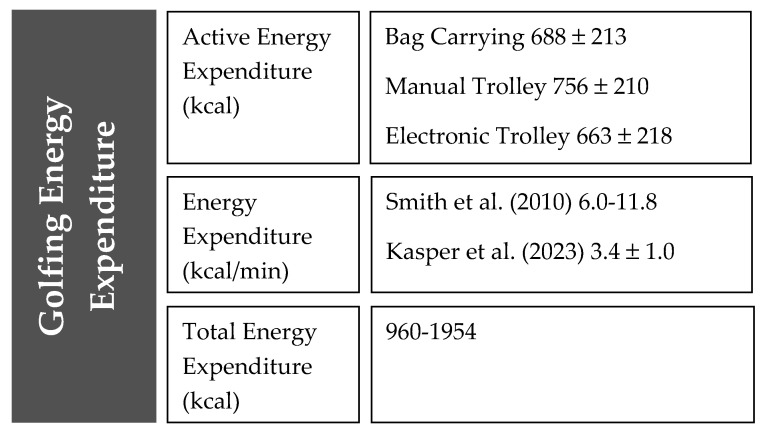
Reported energy demands of playing 18 holes of golf: Different transport modalities. Data Sources: [5,10].

Although exercise energy expenditure is well documented in other sports, the literature is scarce for elite golf performance. Moreover, different methodologies and demands of the sport make comparisons between studies difficult. For example, a professional golfer will play a competitive round of 18 holes with the assistance from a caddy (club carrier), vs. an elite amateur that may play 36 competitive holes of golf while carrying their own bag (US amateur format) and thus creating a larger energy expenditure during training and competition. A recent study by Kasper and colleagues investigated the energy expenditure (EE) for different transportations of clubs (carrying bag, manual trolley and electronic trolley) [10]. The results showed an EE range of 663 kcal–756 kcal per competitive round of golf [10]. These data indicate that EE in elite golf could be 3.4 kcal ± 1.0 kcal/min, which can provide the basis to inform nutritional interventions [10]; more details are presented in Figure 1. In addition to this, the best available literature highlights that golf provides moderate intensity activity for most people at 4.8 metabolic equivalents (METs) [11,12].

## 4. Physical Attributes of Elite Golf: Training Requirements

Golf is viewed as a skill-based sport, in which the refinement of ball striking and putting skills has been given more emphasis than the development of physical fitness [11,12,13]. However, as mentioned previously, the course length and distances to the holes are increasing, and strength training may need to be prioritised to maximise strokes gained off the tee [13,14]. Total driving distance has been considered as either the first or second most important predictor of competitive success [13]. Aerobic fitness should not be ignored however, as evidence suggest that athletes perform better at submaximal workloads when aerobic training regimes are developed [15].

The physiques of golfers have received little attention and more modern players are now realising the transition to a better physique may have performance benefits [14]. The physique of a professional golfer can vary widely and players at the top of the game demonstrate different body shape and composition. Although golf is lower in intensity, higher body fat levels, paired with lower fitness levels, can impair performance outcomes through poorer heat tolerance and higher susceptibility to physical fatigue and an increased risk of injuries [5]. However, the discussion around body composition and physical fitness in golf is beginning to change and physical training programs for golfers are now considered an integral component of an elite player’s regimen [14].

Alvarez and colleagues [14] conducted a study to determine the effect of an 18-week, three-part strength training program focusing on increasing maximal strength in low handicap male golfers. Ten male golfers with a handicap ≤5 were randomly assigned into either a control group (CG) or a treatment group (TG). The CG followed a standard physical conditioning program with a golf specific focus, whereas the TG participated in an 18-week program divided into three parts: maximal strength training (weightlifting), explosive strength training (plyometrics) and golf specific strength training. In addition to regular golf training, this specific program increased maximal strength, explosive strength of lower limbs and driving performance in terms of club head and ball speed [14]. These improvements remained unaltered during the 6-week golf specific training period and following a 5-week detraining period [14]. This demonstrates the potential benefit of strength training as the adaptations from the resistance exercise remained unaltered during a detraining block which could show similarities to an elite golfer experiencing long duration travel commitments. Golfers could potentially benefit from strength training programs if there is a positive transfer of the effects of these programs to driving performance [14].

## 5. Nutrition Considerations for Elite Golf

Over the past decade, there has been an increasing interest in sports nutrition and the impact of dietary strategies, diet quality and ergogenic supplements on training and sports performance across a wide range of sporting codes. These include significant published literature containing position and consensus statements from expert sport science and medical bodies, which include the International Olympic Committee and American College of Sports Medicine. The primary focus of the research has not only been on physical outcomes, with a growing body of work exploring sports and activities that are more cognitive performance based, as well as how nutrition strategies can impact different cognitive aspects from a decision-making and skill acquisition point of view. Despite this, there is still limited literature regarding the nutritional guidelines for golf performance. The next section will highlight how nutrition has the potential to enhance golf performance or outcomes that can potentially correlate to improved golfing outcomes. Although the literature is lacking in specific areas, recommendations of energy needs, macronutrient intakes, hydration status, supplements and the demands of travel for consideration for the elite golfer are summarised in Table 2 below.

### 5.1. Fuelling for Elite Golf—Carbohydrate Needs

Both endogenous and exogenous carbohydrates can improve training and competition in a variety of endurance and high-intensity intermittent sports [31]. It is well established that the consumption of carbohydrates immediately before and during exercise represents an effective strategy to provide exogenous fuel sources to the brain and working muscle [21,32]. During submaximal moderate-intensity exercise of long duration, the pre-event meal and nutrition provided during training/competition become important [21]. General sports nutrition guidelines suggest that the pre-exercise meal should be consumed approximately 1–4 h prior to exercise and should aim to top up muscle and liver glycogen [21,32]. During moderate intensity exercise, it has been suggested that the consumption of 30–60 g of carbohydrates per hour to ensure optimal fuel availability for the working muscle and brain is required [21,32]. Continuous feeding of carbohydrates can help alleviate the decline in blood glucose concentrations and has a beneficial effect on the central nervous system which can enhance exercise performance [21,32]. Since the golf swing utilises both slow and fast twitch fibres and different energy systems between anaerobic and aerobic needs, it reinforces the importance of ingesting carbohydrates during competition [13].

Although the overall intensity of golf is much lower compared to other sports, it is important to consider the distances travelled, which can exceed 10 km per round, the constant repetition of powerful movements (golf swing) and the significant amount of time spent on preparation and the golf course. The combination of these factors can cause both physical and mental fatigue over the course of competition or long training sessions. During a competitive round of golf, blood glucose can significantly decrease by 10–30% without nutritional intake, which can negatively impact focus, decision making and depth perception [33]. A recent study found that during a competitive 18-hole event, individuals that did not consume food in the first half of the competition observed, on average, a 20% decline in blood glucose concentrations, suggesting nutritional intake may be required to mitigate these blood glucose changes [5]. Thompsett and colleagues [34] explored the effects of macronutrient feeding on fatigue and showed that those consuming carbohydrate with or without protein foods demonstrated lower self-reported feelings of fatigue [34]. While blood glucose levels were not measured, it is possible that levels were maintained as a result of the feeding. Despite lower self-reported feelings of fatigue, no differences in performance or alertness were observed between intervention and control groups [34].

The focus of a fuelling strategy that promotes stable blood glucose levels can provide the player with valuable awareness to the importance of correct preparation and maintenance for development [5]. Although elite golf competition may not require popular strategies like carbohydrate loading or the use of multiple transportable carbohydrates to maximise fuel utilisation, it would be important for the elite golfer to consider the pre-event meal and continuous carbohydrate-containing snacks during a competitive event to prevent fatigue and promote high carbohydrate availability for repetitive golf swings.

During player development, it is important to consider different training modalities and hence nutrient needs to match. Training sessions focusing on strength adaptations, plyometrics and mobility work can show a reduction of muscle glycogen by up to 40% and it is suggested that adequate daily carbohydrate intake is required for glycogen repletion during periods of regular training [35,36]. Research suggests a moderate carbohydrate intake of 3–5 g per kg of body weight (BW) per day is recommended for strength athletes [20], and additional carbohydrate requirements should match the context of the athlete’s training schedule. Knowing the energy demands of the sport and training demands could provide a starting point for calculating carbohydrate needs alongside training goals and individual preferences.

### 5.2. Recovery for Elite Golf

To promote optimal recovery from training or competition, carbohydrates, protein, fluids and electrolytes will need to be replaced, otherwise health and performance may be hampered [37]. Each of these are important but we will first focus on protein intake for optimal recovery. Proteins are important for many key functions within the body, including structural proteins, contractile proteins, immunoproteins and regulatory proteins. During the recovery process proteins are broken down into constitute amino acids and the remodelling process allows for damaged proteins to be removed and replaced in response to exercise training [31]. This remodelling process is heightened and extremely sensitive after resistance training, which allows for greater training adaptations depending on the environment cells are placed under from nutrient intake [37].

There is a growing appreciation that strength is essential for optimal golf performance and nutrition can enhance these training adaptations when training regimes are aligned [31]. Protein plays an important role in recovery from a bout of exercise, helping the elite golfer maximise training adaptations, lessen the risk of acquiring an illness and ultimately spending more time on the golf course [31]. When trying to achieve gains in lean mass a key focus for athletes is often to maximally stimulate muscle protein synthesis (MPS) to enhance recovery and promote muscle hypertrophy [19]. Dose response studies have revealed that 20 g of high-quality protein is sufficient to maximally stimulate MPS after resistance exercise in average-weight males [18]. General guidelines for protein intake for exercising individuals is in the range of 1.4–2.0 g/kgBW/day, and individuals undertaking strength training can be twice as high as compared to their sedentary counterparts with a recommended consumption of 1.6–2.2 g/kgBW/day [19]. More recent research has looked at a dose response of protein per meal in older males and suggested that the intake can be set at 0.4 g/kgBW per meal over 4–5 feedings a day, which could further enhance the stimulatory response of protein synthesis [18].

In addition to total protein intakes, the timing and frequency of protein feedings over the day has been shown to enhance recovery [38]. Throughout the day the body can switch between MPS and muscle protein breakdown (MPB), depending on fed and exercise states, and the aim of recovery nutrition would be to spend most of the day in a muscle-building state more so than degradation [38]. Evidence has shown that consuming 20–40 g of protein at a sitting, spread across 4–5 meals per day, is optimal for muscle growth and recovery [19]. The difficulty in achieving optimal protein intake for the elite golfer is the vast differences in body composition and training demands, as total daily protein intake recommendations are aligned with an individual’s needs based on their weight. For example, a 70 kg golfer aiming for 2 g/kgBW/day would need a total of 140 g protein spread evenly across the day, whereas a 100 kg golfer, at the same rate, would need to ingest a total of 200 g of protein across the day, which may be more difficult to achieve [31].

Research reveals that the majority of golfers are likely skewing their protein intake towards the back half of the day, meaning at breakfast insufficient amounts of protein are consumed, followed by limited opportunities to consume portable protein containing foods during the day (food safety concerns on the golf course) and then consuming large amounts of protein in the evening [31]. Regardless of whether recovery from elite golf or training adaptations is the end goal, it would be crucial for the golfer to evenly distribute high-quality protein doses of 20–40 g across the day over 4–5 eating occasions [31]. This would mean a focus on protein in the morning meal, during the day on the golf course, evening and potentially before sleep to promote maximal protein synthesis, training adaptations and recovery from competition.

### 5.3. Nutrition Considerations—Between Seasons and Competition for Elite Golf

An elite golfer’s training and competition schedule can be long and demanding, spending most weeks on the road travelling between tournaments. During periods of competition or other stages of the season (pre-season or off season), a golfer’s training load will fluctuate and therefore nutritional periodisation should be considered. This concept incorporates matching food/energy intake with energy demands on higher training or competition days and lower food/energy intake on non-training days where energy demands are lower. For an elite golfer, this concept would mean matching energy intake to the demands of travel or off-season training goals, i.e., for increasing muscle mass.

When considering nutrition periodisation and how physical demands change throughout an entire season (pre, during and off season), energy availability must be considered. Energy availability (EA) reflects the difference between energy intake and exercise energy expenditure in relation to fat-free mass (FFM) [39]. Optimal energy availability in the literature demonstrates energy intake between 40–45 kcal/kgFFM/day, depending on gender (males > 40 kcal and females > 45 kcal) and training status [17]. If training demands are not considered and inadequate energy is consumed, this can lead to low energy availability (LEA). Extended periods of LEA, with intakes of <30 kcal/kgFFM/day, have been shown to lead to endocrine and metabolic alterations affecting health and performance [39]. Health effects of problematic LEA can include menstrual dysfunction, poor bone health, endocrine alterations, diminished growth and development, psychological, cardiovascular and gastrointestinal issues and poor immunological response [17].

Further upon this, problematic LEA can lead to relative energy deficiency in sport (RED-S) and affect athletic performance by increasing injury risks, decreasing training response, impairing judgement and co-ordination, increasing irritability and depression and decreasing muscle strength and endurance [17]. These are all issues an elite golfer needs to consider when aiming to prevent LEA and complications associated with problematic LEA such as RED-S. By carefully periodising nutrition intake to reflect the training/competition demands of the season and travel needs, this can promote optimal energy availability for health and performance [17].

Between competition and off season, golfers may benefit by adapting training sessions to reflect their increasing strength, power and muscle mass. While the energy costs of synthesising new tissue and the mechanisms associated with hypertrophy are extremely difficult to measure, an additional intake/surplus of 300–500 kcal/day (1500–2000 kJ/day) is widely accepted to help with mass gain [16]. Recent literature has also described an energy availability amount of >45 kcal/kgFFM/day as sufficient to enhance muscle hypertrophy and growth alongside other dietary strategies [37]. It is important to consider that if excess energy is consumed without an appropriate training program, fat mass vs. muscle mass gain is likely to occur. Hence, the close monitoring and response to changes in body composition and functional capacity need to be considered to personalise the intervention [16].

### 5.4. Hydration Status and Golf Performance

Tournament conditions can vary considerably depending on season and location. Numerous competitions are played in the summer months and often in the hottest part of the day, illustrating the importance of hydration. Dehydration can potentially affect exercise performance by increasing core body temperature, cardiovascular strain and additional glycogen utilisation, impairing cognitive performance [22,40]. Dehydration of >2% BW has consistently been shown to impair endurance performance and mild dehydration of ≥1–2% BW has been shown to impair sport-specific cognitive performance [22,41].

Both voluntary fluid intake and climate alterations are the main contributors to modifications in an individual’s hydration status [5]. Any onset of cognitive motor dysfunction during golf will impact the ability to select the correct shot type and the execution of the golf swing [5]. The locomotor speeds reported from the American College of Sports Medicine (2007) suggest that travelling at 5–6 km/h can produce a sweat rate of 400 mL/h, which is similar to golf course demands [22]. It is imperative that a fluid intake plan be developed for players to reduce the risk and effects of dehydration and to minimise fluid loss to no greater than 1% of body mass to ensure cognitive function is not impaired [22].

Magee and colleagues [42] assessed the hydration status of 15 elite collegiate male golf athletes with handicaps ranging from 0 to +3. More than 40% of the participants were dehydrated pre-exercise and 60% were dehydrated post-exercise [42]. The mean number of total strokes taken to complete an 18-hole competitive round was significantly higher in players that were dehydrated before commencing competition at 79.5 ± 2.1 strokes compared to euhydrated players who used 75.7 ± 3.9 strokes [42]. Similarly, Smith and colleagues (2012) observed negative impacts on golf performance following an acute mild dehydration [23]. The results showed a significant difference in overall distance achieved using a 9-iron club (9i) in the dehydrated state (114.6 m ± 12.9 m) compared to the euhydrated state (128.6 ± 8.8 m) [23]. Performance for accuracy was also negatively impacted by the dehydrated state with a significantly higher level of inaccuracy for the dehydrated players (7.9 ± 2.0 m) compared to the euhydrated players (4.1 ± 0.8 m). Although other parameters were tested it should be acknowledged that the 9i club would typically be associated with the smallest inaccuracy range compared to the longer and less lofted golf clubs [23].

A limitation for both studies was that hydration status was only assessed immediately before and/or after competition with no repeated measures of average hydration levels. This information could have provided a more accurate representation of hydration status over the prolonged duration of a competitive round of golf. Furthermore, some of these studies were conducted in the UK/Ireland and may not correlate to other environmental settings such as warmer climates where hydration status may play a more crucial role. However, the above findings show that mild dehydration can affect golf specific performance in terms of both distance and accuracy and the individual would need to consider the importance of hydration.

Players should be encouraged to manage hydration status throughout the entire round by commencing competition euhydrated and having a personalised fluid intake plan for competition that prevents dehydration of >1% BW reduction from baseline BW over the round [23]. The consumption of beverages containing electrolytes and carbohydrates should ensure adequate hydration, offsetting the effects of fatigue on cognitive performance [23]. Rehydration solutions, such as sports drinks, often contain fluids, electrolytes and or carbohydrates to provide additional fuel [43]. The additional carbohydrates in these beverages will also provide important fuel for the work required during competitive golf and therefore the use of such fluids could provide a convenient fuel source that can combat dehydration and potentially enhance on-course performance.

## 6. Supplements for Golf Performance

The traditional development of ergogenic approaches in the game of golf have focused on modifying clubs and other pieces of equipment intended to help improve performance. Considering the physical and cognitive challenges brought forth by golf, nutritional supplements may also impact performance, but minimal research has been undertaken in this area to date. The limited available literature on supplements that have been demonstrated to have some potential benefit in relation to golf performance are presented below.

### 6.1. Caffeine

Caffeine is arguably one of the most popular supplements and is known for its effects of being an adenosine receptor antagonist, reducing levels of perceived exertion, pain perception and the ability to increase motor unit recruitment in active muscles [44]. Athletes are always adopting new strategies to enhance performance, and most are aware of caffeine’s ergogenic benefits with approximately 75% of athletes consuming caffeine around competitions [45]. Golf is seen as a tactical sport, and it is not surprising that there is evidence to suggest caffeine is a supplement that can enhance golf performance. Competitive golf contains high cognitive loads, critical shot making decisions, hand–eye coordination and high levels of motor skills [5]. Mental fatigue may affect the ability to select the correct club, shot type and execution of the shot, whereas physical fatigue may affect the performance of the golf swing [46].

Mumford and colleagues investigated the effects of a caffeine-containing supplement on golf performance and fatigue during a competitive 36-hole tournament [24]. Twelve male golfers (handicap 3–10) participated in a double-blind controlled, crossover design study and played 18 holes on two consecutive days with each assigned to a caffeine-containing group (1.9 ± 0.3 mg/kg) or a matched placebo. Caffeine (CAF) or the placebo (PLA) were ingested 25–35 min before the initiation of the 18-hole competitions, and participants were administered a second dose after the completion of 9 holes (total CAF/round 3.8 ± 0.6 mg/kg). At the halfway mark, participants were also provided with a standardised meal aiming to offset the decline in blood glucose levels (340 kcal, 42 g carbohydrates, 12 g fat, 24 g protein). While this study assessed a range of different variables, golf-specific performance outcomes are of primary interest in this review. The results showed that no substantial differences were recorded in the number of fairways hit, putts per round, shots hit out of bounds, sand shots, sand save percentages and first putt distances missed between the conditions [24]. The total score (PLA = 79.4 ± 9.1 vs. CAF = 76.9 ± 8.1), greens in regulation (PLA = 6.9 ± 4.6 vs. CAF = 8.7 ± 3.4) and drive distance (PLA = 233.3 m ± 32.5 vs. CAF = 239.9 ± 33.8 m) were statistically significantly better under the CAF conditions compared to the PLA [24]. This study found a decline in the measures of self-reported energy levels and an increased perception of fatigue throughout a competitive round of golf. Caffeine supplementation was able to significantly attenuate the perception of fatigue during the round, which may have contributed to the findings of improved measures in golf performance [24]. This study demonstrated that caffeine intake at doses in the range of 3–5 mg/kgBW could minimise golf-specific fatigue and hence, contribute to enhanced golf performance by improving accuracy and drive distance [24].

The combination of caffeine with glucose has also been shown to improve aspects of cognitive performance to a greater extent than when consumed separately for golfers. Stevenson and colleagues (2009) investigated the effects of an isotonic sports drink containing both carbohydrate (CHO) and caffeine (6.4 g CHO + 16 mg CAF/100 mL) or a no-energy, flavour-matched placebo consumed as a bolus (5 ml/kg) prior to playing an 18-hole game of golf and an additional serving (2.5 mL/kg) at holes 6 and 12, respectively. Results showed that 2 m putting performance was significantly greater when caffeine and carbohydrate were consumed compared to the placebo over the final six holes of the round with 70% of successful putts holed by the intervention group against 50% for the control group [47]. In the 5 m putting performance, the percentage of successful putts made were significantly greater in the CAF + CHO trial compared with the placebo group across the final six holes (40% vs. 25%). The number of successful putts for the 18-hole trial overall was also significantly higher in the CAF + CHO trial compared to the placebo group [47]. This study showed that during the final six holes, where the final leader of the tournament is usually determined, the percentage of successful putts was higher in the CAF + CHO trial compared to the placebo [47]. These improvements were likely to be attributable to the reduction in fatigue over a competitive round of golf [47].

While it cannot be determined how much of a difference in performance benefits seen could be attributed to reduced carbohydrate availability in the placebo group or the placebo group abstaining from their usual caffeine intake and its detriment on cognitive function [47]; this is still a significant finding. It is also important to consider that a professional golfer would have spent significant amounts of time attempting to perfect their putting stroke and there could potentially be less room for improvement compared to the recreational golfers who partook in this study, irrespective of intervention.

### 6.2. Creatine Monohydrate

As this review has considered training adaptations for enhancing golf performance, the potential role of creatine monohydrate (CM) supplementation will be discussed, as studies have previously reported its ergogenic benefits in anaerobic and strength-based exercises. Creatine monohydrate can enhance athlete performance in sports involving repeated bouts of high-intensity exercise, as well as chronic adaptations of training programs based on these characteristics (e.g., resistance or interval training) leading to greater gains in lean mass and muscular strength and power [26,48].

Ziegenfuss et al. (2015) looked at the effect of a popular golfing supplement, Strong Drive (SD), which incorporated a mixture of CM (5 g), coffee extract (50 mg), calcium fructoborate and vitamin D [25] aiming to assess golf drive performance using a double-blind placebo-control trial. Participants were required to consume SD and undertake a strength training regime over a 30-day period. The participants were instructed to consume SD or placebo twice per day for the first two weeks and during the final two weeks ingested only one serving per day, which is similar to slow loading protocols for CM [25]. The primary outcome variables of interest in the study were peak and average club head speed, ball speed and average and best distance for each club [25].

The results showed that participants supplementing with SD over the 4-week period increased their overall total driving distance and tended to improve their average driver distance [25]. The average driving distance increased significantly from 269.9 ± 18.5 to 283.5 ± 23.1 yards (y), adding a total of 13.6 ± 29.0 y in driving distance. Both groups experienced significant improvements in bench press strength while the SD group also experienced significant improvements in peak power and peak velocity production [25]. These findings are highly relevant because of the study’s ‘free living’ approach, which is applicable to many athletes who play golf and may not consider the importance of dietary intake or supplementation [25]. However, the interpretation of results is limited to recreational golfers and may not reflect the practices of elite golfers. Nonetheless, these results suggest that golfers should consider the potential benefits of CM supplementation alongside strength training when aiming to increase their total driving distance.

## 7. Nutritional Issues for the Elite Travelling Golfer

Elite golfers can spend most of their annual competition schedule travelling interstate or internationally to play major tournaments. This involves great perturbation and stress on an athlete as it tends to affect optimal baseline states [49]. Flying between continents across multiple time zones can induce jet lag or circadian desynchronisation (changes in body clock rhythms), which can produce symptoms such as fatigue, sleep–wake disturbances, mood changes, bowel disturbance and impaired cognitive function [50]. There is evidence of the importance of circadian rhythmicity for athletic performance, with delineating effects for various aspects such as strength, anaerobic and aerobic performance [51]. The desynchronisation of the circadian rhythm caused by rapid air travel across multiple time zones has the potential to affect athletic performance over a 24-h period [51].

Travelling for competition also often changes the eating behaviour and food choices of the athlete and can compromise nutrition goals and practices focused on competition [52]. Athletes frequently snack on inappropriate food choices away from home and convenient stops such as local restaurants can promote further purchases of snacks or inappropriate ‘convenient’ meals [52]. Athletes travelling to different countries face many challenges relating to food choices, such as unfamiliar foods, unavailability of common foods and different eating occasions due to time zones and jet lag [49]. Adequate food choices may be available at elite level competitions where athletes stay in luxurious hotels or residential environments; however, in smaller competitions, athletes may be less advantaged, as access to common foods will be more difficult and will affect food choice, delivery and environment [49]. The largest challenges that the travelling golfer faces is the need to ensure optimal health and familiar food intake promoting optimal performance.

### Nutrition Strategies for Travel

The travelling golfer should aim to eat similar foods to home and, where possible, avoid any variations in their usual food intake to lower the risk of gastrointestinal (GI) distress, food-borne illness and any other dietary-related problems [53]. Prior to travelling overseas, golfers should be provided with the opportunity to become familiar with the food and culture of the destination, any challenges associated eating away from home, understand the food preparation and storage facilities which will be available to them at their accommodation (e.g., refrigeration and kitchen equipment) and reinforce the importance of good hygiene practices and nutritional strategies [53].

Travelling in unfamiliar environments can also directly affect an individual’s immune system and integrity [13]. Adequate vitamin D levels have been shown to reduce the incidence of upper respiratory tract infections (URTI) [54]. A recent study found that higher levels of vitamin D can have significant effects on the immune system above the guideline level of 25 OHD > 30 ng/mL [30]. This study of 14,108 participants showed that individuals with levels <30 ng/mL had a 58% higher chance of getting an acute respiratory infection [30]. Due to an elite golfer’s schedule and travel demands, it may be a consideration to assess their baseline vitamin D level and supplement vitamin D, if required, to ensure levels are >30 ng/mL, especially during tournaments and travel where sunlight exposure may be limited [13].

## 8. Future Directions for Research

There is an abundance of opportunity warranting investigation in the nutrition evidence base for golf-specific performance. Researchers should begin to look at the dietary intake and energy expenditure of elite golfers to determine optimal energy availability to promote fuelling, recovery and optimal health and performance, especially if enduring large travel demands. It is stated in the literature that problematic LEA, leading to RED-S, can have a significant impact on immune cells, therefore increasing the likelihood of infections and illness severity and duration [17]. Understanding current energy and nutrient intake, as well as the energy expenditure of golfers, will help guide advice to athletes to avoid problematic LEA and RED-S.

Upon promoting optimal energy availability, carbohydrate needs specific for competitive golf could be explored. Preventing hypoglycaemia and any potential decline in cognitive function associated with low blood glucose levels may help the elite golfer perform more consistently on course and dictate fuelling strategies [55]. Currently available technology, such as continuous glucose monitors, may allow practitioners and athletes to gain feedback on training or competition sessions to help create plans or adjust in response to undesirable glucose fluctuations during training and competition [55].

Hydration status is another important consideration given evidence shows that as little as 1% dehydration can impair cognitive performance and potentially golf-related performance [22]. However, more work needs to be undertaken to explore the personalisation of hydration strategies which should: account for individual sweat rates and incorporate different climates across the globe, course topology and long competitive demands, including warm up and cool down, all of which can lead to significant heat exposure and the potential for dehydration.

The literature on the efficacy of ergogenic aids in golf performance is scarce. There have been a few supplements of interest demonstrating enhanced golf performance; with caffeine supplementation delaying the onset of fatigue [24] and creatine monohydrate positively impacting short intense repetitive movements and cognitive function [25]. However, this literature is limited to studies on recreational golfers and future research should aim to publish data within the elite population as their capacity for further improvement may not be as great. Assessing supplement knowledge and awareness of batch-tested products has not been explored to the authors’ knowledge and educating golfers on the potential risks of contamination can be of value. Lastly, future research should explore the potential efficacy of other dietary supplements, such as gut modulating products: prebiotics and or probiotics (plus many others), to improve the resilience of the gut microbiome as well as enhance cognitive performance via the microbiota and gut–brain axis [56]. Additionally, L-Menthol can be trialled in warm climates where heat tolerance could be an issue for a golfer and the potential cooling effects that menthol has on preventing feelings of fatigue in the heat explored [29].

Finally, the continuing work in travel nutrition should be prioritised within the elite golf population, given the nature of the sport and the competition circuit. The exploration of the effectiveness of travel-related food, sleep and hydration strategies should be implemented to reduce fatigue for golfers travelling across multiple time zones throughout the competitive season.

## 9. Conclusions

This review highlights the importance of nutrition considerations for elite golfers. Currently, there is a lack of published data on nutritional intakes and macronutrient requirements of elite golfers, with a small body of work focusing on golf-specific nutrition-related studies aiming to enhance training adaptations, carbohydrate needs, hydration status, caffeine protocols and use of creatine monohydrate. It demonstrates that appropriate the composition and timing of meals, euhydration prior to the commencement of competition and training and effective rehydration strategies, as well as supplementation with caffeine and creatine monohydrate, may all lead to improvements in golf performance outcomes, via both physiological and cognitive effects. There is a need for further research on elite golfers to explore: energy availability concerns, macronutrient specific strategies to enhance golf performance, individualised hydration strategies for different climates, supplementation protocols and relating these into the travel demands that an elite golfer endures across a long competitive season.

## Figures and Tables

**Table 1:** Sample of an elite PGA professional golfer’s 2022–2023 competition schedule.

Date	Golf Tour	Tournament	Position
Jul. 7–10th	DP World Tour	Scottish Open (Scotland)	T 10th
Jul. 14–17th	PGA Tour	The Open Championship (United Kingdom)	1st
Aug. 11–14th	PGA Tour	Fed Ex St Jude Championship (USA)	T 13th
Aug. 26–29th	PGA Tour	The PGA Championship (USA)	20th
Sep. 3–5th	LIV Tour	LIV Golf: Invitational Boston (USA)	T 4th
Sep. 17–19th	LIV Tour	LIV Golf: Invitational Chicago (USA)	1st
Oct. 7–9th	LIV Tour	LIV Golf: Invitational Bangkok (Thailand)	41st
Oct. 14–16th	LIV Tour	LIV Golf: Invitational Jeddah (Saudi Arabia)	T 21st
Nov. 24–27th	DP World Tour	AUS PGA Championship (Australia)	1st
Feb. 2–5th	Asian Tour	Saudi International (Saudi Arabia)	M/C
Feb. 25–27th	LIV Tour	LIV Invitational Golf Mayakoba (Mexico)	T 5th
Mar. 18–20th	LIV Tour	LIV Invitational Golf Tucson (USA)	T 24th
Apr. 1–3rd	LIV Tour	LIV Invitational Golf Orlando (USA)	T 26th
Apr. 6–9th	PGA Tour	The Masters Tournament (USA)	T 34th
Apr. 21–23rd	LIV Tour	LIV Golf Adelaide (AUS)	T 3rd
Apr. 28–30th	LIV Tour	LIV Golf Singapore (Singapore)	T 6th
May. 13–15th	LIV Tour	LIV Golf Tulsa (USA)	T 2nd
May. 18–21st	PGA Tour	The PGA Championship (USA)	T 9th
May. 27–29th	LIV Tour	LIV Golf Washington (USA)	T 9th
Jun. 15–18th	PGA Tour	The US Open (USA)	4th
Jun. 30–Jul. 2nd	LIV Tour	LIV Golf Valderrama (Spain)	T 12th

Source: Table created by authors from data available on https://www.pgatour.com/player/35891/cameron-smith (accessed on 30 August 2023).

**Table 2:** Summary of practical guidelines for nutritional consideration in elite golf.

Consideration	Comments	Reference
Energy Intake	Aim for optimal energy availability for season-specific energy demands. Energy availability 40–45 kcal/kgFFM/day to benefit health and performance outcomes. If an increase in muscle mass is desired than an energy surplus of 300–500 kcal per day (1500–2000 kJ) is required. Needs to be individualised to the training/competitive context.	[16,17]
Protein	No specific guidelines for protein intake and golf performance exist. If the athlete is undertaking a resistance training program focusing on enhancing strength outcomes, then consider 1.4–2.0 g/kgBW/day or per meal dose of 0.4 g/kgBW of quality protein sources distributed evenly throughout the day over 4–5 meals.	[18,19]
Carbohydrate	No specific guidelines for carbohydrate intake and golf performance exist. Energy demands are approximately 700 kcal a competitive round with carbohydrate being the main source of energy. Carbohydrate intake should focus on supporting training and competition demands. If undertaking resistance training to facilitate strength gains, then a range of 3–5 g/kgBW/day depending on context would be suitable. Carbohydrate feeding during competitive rounds should be considered to offset blood glucose decline and improve cognitive function.	[10,13,20,21]
Hydration Status	Elite golfers should look to maintain a euhydrated state throughout the entire day/competition if possible, or if not possible, commence competition/training in a euhydrated state and minimise fluid loss to no more than 1% loss of BW. Beverages containing electrolytes and carbohydrates should be consumed to ensure adequate hydration to optimise cognitive function and remain euhydrated.	[13,22,23]
Supplement Use	Caffeine: No specific guidelines on caffeine use for golf performance exist. However, 2–5 mg/kgBW, consumed 30 min prior to training/competition, followed by top-ups over a game have been shown to help reduce perceived fatigue and enhance golf performance [24].Creatine monohydrate: No specific guidelines for golf performance exist. However, evidence suggests that to enhance resistance training adaptations daily dosage protocols of 5 g/day for at least 1 month may be sufficient in increasing muscle creatine stores [25].	[13,24,25,26,27,28,29]
Travel Nutrition	Elite golfers should plan nutritional strategies prior to leaving for competition. Education and familiarisation with culture and environment can help. Education on maintaining circadian rhythm during the demands of travel can be beneficial. Consider the use of vitamin D supplementation to keep levels >30 ng/mL and reduce risk of upper respiratory tract infections [30].	[13,30,31,32]

## References

[1] Ancient T.R. 2020 GB&I Golf Participation Report. https://www.randa.org/TheRandA/AboutTheRandA/DownloadsAndPublications.

[2] Sport Dietitians Australia Food for Your Sport—Golf Sports Dietitians Australia. https://www.sportsdietitians.com.au/wp-content/uploads/2020/09/FFYS_Golf_2016.pdf.

[3] TPG Association PGA Tour Tournament Schedule. https://www.pgatour.com/schedule.com.

[4] Parkkari J., Natri A., Kannus P., Mänttäri A., Laukkanen R., Haapasalo H., Nenonen A., Pasanen M., Oja P., Vuori I. (2000). A controlled trial of the health benefits of regular walking on a golf course. Am. J. Med..

[5] Smith M.F. (2010). The Role of Physiology in the Development of Golf Performance. Sports Med..

[6] Kosendiak J., Naglak F., Kosendiak J. (2007). Evaluation of the Polish National team Junior Golf Players’ anaerobic function and motor capacity. Stud. Phys. Cult. Tour..

[7] Stauch M., Liu Y., Giesler M., Lehmann M. (1999). Physical activity level during a round of golf on a hilly course. J. Sports Med. Phys. Fitness.

[8] Sell T.C., Tsai Y.S., Smoliga J.M., Myers J.B., Lephart S.M. (2007). Strength, flexibility, and balance characteristics of highly proficient golfers. J. Strength Cond. Res..

[9] Keogh J.W., Marnewick M.C., Maulder P.S., Nortje J.P., Hume P.A., Bradshaw E.J. (2009). Are anthropometric, flexibility, muscular strength, and endurance variables related to clubhead velocity in low- and high-handicap golfers?. J. Strength. Cond. Res..

[10] Kasper A.M., O’Donnell A., Langan-Evans C., Jones A., Lindsay A., Murray A., Close G.L. (2023). Assessment of activity energy expenditure during competitive golf: The effects of bag carrying, electric or manual trolleys. Eur. J. Sport. Sci..

[11] Luscombe J., Murray A.D., Jenkins E., Archibald D. (2017). A rapid review to identify physical activity accrued while playing golf. BMJ Open.

[12] Murray A.D., Daines L., Archibald D., Hawkes R.A., Schiphorst C., Kelly P., Grant L., Mutrie N. (2017). The relationships between golf and health: A scoping review. Br. J. Sports Med..

[13] Zoffer M. (2022). Competitive Golf: How Longer Courses Are Changing Athletes and Their Approach to the Game. Nutrients.

[14] Alvarez M., Sedano S., Cuadrado G., Redondo J.C. (2012). Effects of an 18-week strength training program on low-handicap golfers’ performance. J. Strength Cond. Res..

[15] Ramos-Jiménez A., Hernández-Torres R.P., Torres-Durán P.V., Romero-Gonzalez J., Mascher D., Posadas-Romero C., Juárez-Oropeza M.A. (2008). The Respiratory Exchange Ratio is Associated with Fitness Indicators Both in Trained and Untrained Men: A Possible Application for People with Reduced Exercise Tolerance. Clin. Med. Circ. Respir. Pulm. Med..

[16] Slater G.J., Dieter B.P., Marsh D.J., Helms E.R., Shaw G., Iraki J. (2019). Is an Energy Surplus Required to Maximize Skeletal Muscle Hypertrophy Associated with Resistance Training. Front. Nutr..

[17] Mountjoy M., Sundgot-Borgen J., Burke L., Ackerman K.E., Blauwet C., Constantini N., Lebrun C., Lundy B., Melin A., Meyer N. (2018). IOC consensus statement on relative energy deficiency in sport (RED-S): 2018 update. Br. J. Sports Med..

[18] Moore D.R., Churchward-Venne T.A., Witard O., Breen L., Burd N.A., Tipton K.D., Phillips S.M. (2015). Protein ingestion to stimulate myofibrillar protein synthesis requires greater relative protein intakes in healthy older versus younger men. J. Gerontol. A Biol. Sci. Med. Sci..

[19] Witard O.C., Wardle S.L., Macnaughton L.S., Hodgson A.B., Tipton K.D. (2016). Protein Considerations for Optimising Skeletal Muscle Mass in Healthy Young and Older Adults. Nutrients.

[20] Slater G., Phillips S.M. (2011). Nutrition guidelines for strength sports: Sprinting, weightlifting, throwing events, and bodybuilding. J. Sports Sci..

[21] Burke L.M., Hawley J.A., Wong S.H., Jeukendrup A.E. (2011). Carbohydrates for training and competition. J. Sports Sci..

[22] Sawka M.N., Burke L.M., Eichner E.R., Maughan R.J., Montain S.J., Stachenfeld N.S. (2007). American College of Sports Medicine position stand. Exercise and fluid replacement. Med. Sci. Sports Exerc..

[23] Smith M.F., Newell A.J., Baker M.R. (2012). Effect of acute mild dehydration on cognitive-motor performance in golf. J. Strength Cond. Res..

[24] Mumford P.W., Tribby A.C., Poole C.N., Dalbo V.J., Scanlan A.T., Moon J.R., Roberts M.D., Young K.C. (2016). Effect of Caffeine on Golf Performance and Fatigue during a Competitive Tournament. Med. Sci. Sports Exerc..

[25] Ziegenfuss T.N., Habowski S.M., Lemieux R., Sandrock J.E., Kedia A.W., Kerksick C.M., Lopez H.L. (2015). Effects of a dietary supplement on golf drive distance and functional indices of golf performance. J. Int. Soc. Sports Nutr..

[26] Volek J.S., Rawson E.S. (2004). Scientific basis and practical aspects of creatine supplementation for athletes. Nutrition.

[27] Talanian J.L., Spriet L.L. (2016). Low and moderate doses of caffeine late in exercise improve performance in trained cyclists. Appl. Physiol. Nutr. Metab..

[28] Hultman E., Söderlund K., Timmons J.A., Cederblad G., Greenhaff P.L. (1996). Muscle creatine loading in men. J. Appl. Physiol..

[29] Flood T.R., Waldron M., Jeffries O. (2017). Oral L-menthol reduces thermal sensation, increases work-rate and extends time to exhaustion, in the heat at a fixed rating of perceived exertion. Eur. J. Appl. Physiol..

[30] Monlezun D.J., Bittner E.A., Christopher K.B., Camargo C.A., Quraishi S.A. (2015). Vitamin D status and acute respiratory infection: Cross sectional results from the United States National Health and Nutrition Examination Survey, 2001–2006. Nutrients.

[31] GClose L., Pugh J.N., Morton J. (2017). Nutrition for Golf. Routledge International Handbook of Golf Science.

[32] Jeukendrup A.E. (2017). Periodized Nutrition for Athletes. Sports Med..

[33] Broman G., Johnsson L., Kaijser L. (2004). Golf: A high intensity interval activity for elderly men. Aging Clin. Exp. Res..

[34] Thompsett D.J., Vento K.A., Der Ananian C., Hondula D., Wardenaar F.C. (2022). The effects of three different types of macronutrient feedings on golf performance and levels of fatigue and alertness. Nutr. Health.

[35] Robergs R.A., Pearson D.R., Costill D.L., Fink W.J., Pascoe D.D., Benedict M.A., Lambert C.P., Zachweija J.J. (1991). Muscle glycogenolysis during differing intensities of weight-resistance exercise. J. Appl. Physiol..

[36] Camera D.M., Edge J., Short M.J., Hawley J.A., Coffey V.G. (2010). Early time course of Akt phosphorylation after endurance and resistance exercise. Med. Sci. Sports Exerc..

[37] Beck K.L., Thomson J.S., Swift R.J., von Hurst P.R. (2015). Role of nutrition in performance enhancement and postexercise recovery. Open Access J. Sports Med..

[38] Areta J.L., Burke L.M., Ross M.L., Camera D.M., West D.W., Broad E.M., Jeacocke N.A., Moore D.R., Stellingwerff T., Phillips S.M. (2013). Timing and distribution of protein ingestion during prolonged recovery from resistance exercise alters myofibrillar protein synthesis. J. Physiol..

[39] Loucks A.B. (2013). Energy Balance and Energy Availability. The Encyclopaedia of Sports Medicine.

[40] Ganio M.S., Armstrong L.E., Casa D.J., McDermott B.P., Lee E.C., Yamamoto L.M., Marzano S., Lopez R.M., Jimenez L., Le Bellego L. (2011). Mild dehydration impairs cognitive performance and mood of men. Br. J. Nutr..

[41] Cheuvront S.N., Kenefick R.W. (2014). Dehydration: Physiology, assessment, and performance effects. Compr. Physiol..

[42] Magee P.J., Gallagher A.M., McCormack J.M. (2017). High Prevalence of Dehydration and Inadequate Nutritional Knowledge Among University and Club Level Athletes. Int. J. Sport. Nutr. Exerc. Metab..

[43] Maughan R.J., Burke L.M., Dvorak J., Larson-Meyer D.E., Peeling P., Phillips S.M., Rawson E.S., Walsh N.P., Garthe I., Geyer H. (2018). IOC consensus statement: Dietary supplements and the high-performance athlete. Br. J. Sports Med..

[44] Doherty M., Smith P.M. (2005). Effects of caffeine ingestion on rating of perceived exertion during and after exercise: A meta-analysis. Scand. J. Med. Sci. Sports.

[45] Aguilar-Navarro M., Muñoz G., Salinero J.J., Muñoz-Guerra J., Fernández-Álvarez M., Plata M.D., Del Coso J. (2019). Urine Caffeine Concentration in Doping Control Samples from 2004 to 2015. Nutrients.

[46] Doan B.K., Newton R.U., Kraemer W.J., Kwon Y.H., Scheet T.P. (2007). Salivary cortisol, testosterone, and T/C ratio responses during a 36-hole golf competition. Int. J. Sports Med..

[47] Stevenson E.J., Hayes P.R., Allison S.J. (2009). The effect of a carbohydrate-caffeine sports drink on simulated golf performance. Appl. Physiol. Nutr. Metab..

[48] Rawson E., Persky A. (2007). Mechanisms of muscular adaptations to creatine supplementation. Int. Sport. J..

[49] Halson S.L., Burke L.M., Pearce J. (2019). Nutrition for Travel: From Jet lag To Catering. Int. J. Sport. Nutr. Exerc. Metab..

[50] Rajaratnam S.M., Howard M.E., Grunstein R.R. (2013). Sleep loss and circadian disruption in shift work: Health burden and management. Med. J. Aust..

[51] Leatherwood W.E., Dragoo J.L. (2013). Effect of airline travel on performance: A review of the literature. Br. J. Sports Med..

[52] Burke L.M., Deakin V. (2015). Medical and Nutritional Issues for the Travelling Athlete. Clinical Sports Nutrition.

[53] Burke L.M., Deakin V. (2015). Immunity, Infective Illness and Injury. Clinical Sports Nutrition.

[54] Pyne D.B., Gleeson M., McDonald W.A., Clancy R.L., Perry C., Fricker P.A. (2000). Training strategies to maintain immunocompetence in athletes. Int. J. Sports Med..

[55] Bowler A.M., Whitfield J., Marshall L., Coffey V.G., Burke L.M., Cox G.R. (2023). The Use of Continuous Glucose Monitors in Sport: Possible Applications and Considerations. Int. J. Sport. Nutr. Exerc. Metab..

[56] Cooke M.B., Catchlove S., Tooley K.L. (2022). Examining the Influence of the Human Gut Microbiota on Cognition and Stress: A Systematic Review of the Literature. Nutrients.

